# Notification that new names of prokaryotes, new combinations, and new taxonomic opinions have appeared in volume 74, part 7 of the IJSEM

**DOI:** 10.1099/ijsem.0.006494

**Published:** 2024-11-05

**Authors:** Aharon Oren, Markus Göker

**Affiliations:** 1 The Institute of Life Sciences, The Hebrew University of Jerusalem, The Edmond J. Safra Campus, 9190401 Jerusalem, Israel; 2 Leibniz Institute DSMZ – German Collection of Microorganisms and Cell Cultures, Inhoffenstrasse 7B, 38124 Braunschweig, Germany

This listing of names of prokaryotes published in a previous issue of the *International Journal of Systematic and Evolutionary Microbiology* (IJSEM) is provided as a service to microbiology to assist in the recognition of new names and new combinations. This procedure was proposed by the Judicial Commission [1]. The names given herein are listed according to the rules of priority (i.e., time and date of publication of the articles on the journal’s platform and the order of valid publication of names in the original articles) [2].

**Table:** 

Order of article publication	Name/authors	Proposed as	Article no.	Page no.
1	*Nocardioides bizhenqiangii* Zhou *et al*. 2024	sp. nov.	006437	7
1	*Nocardioides renjunii* Zhou *et al*. 2024	sp. nov.	006437	8
2	*Lacticaseibacillus zeae* subsp. *zeae* (Dicks *et al*. 1996 *ex* Kuznetsov 1959) Grabner F *et al.* 2024	comb. nov. [basonym: *Lactobacillus zeae* (*ex* Kuznetsov 1959) Dicks *et al.* 1996]	006441	10
2	*Lacticaseibacillus zeae* subsp. *silagei* Grabner F *et al.* 2024	subsp. nov.	006441	10
2	*Lacticaseibacillus parahuelsenbergensis* Grabner F *et al.* 2024	sp. nov.	006441	11
2	*Lacticaseibacillus styriensis* Grabner F *et al.* 2024	sp. nov.	006441	11
3	*Streptococcus raffinosi* Nguyen *et al.* 2024	sp. nov.	006442	5
4	*Elongatibacter* Zhang *et al.* 2024	gen. nov.	006440	6
4	*Elongatibacter sediminis* Zhang *et al.* 2024	sp. nov.	006440	6
5	*Chitinophaga pollutisoli* Lee *et al.* 2024	sp. nov.	006447	7
6	*Spartinivicinus poritis* Sajid *et al.* 2024	sp. nov.	006444	7
7	*Promethearchaeum* Imachi *et al.* 2024	gen. nov.	006435	8
7	*Promethearchaeum syntrophicum* Imachi *et al.* 2024*	sp. nov.	006435	8
7	*Promethearchaeaceae* Imachi *et al.* 2024	fam. nov.	006435	9
7	*Promethearchaeales* Imachi *et al.* 2024	ord. nov.	006435	9
7	*Promethearchaeia* Imachi *et al.* 2024	class. nov.	006435	9
7	*Promethearchaeota* Imachi *et al.* 2024	phyl. nov.	006435	9
7	*Promethearchaeati* Imachi *et al.* 2024	regn. nov.	006435	9
8	*Acinetobacter pecorum* Pallen 2024	sp. nov.	006445	1
8	*Arthrobacter gallicola* Pallen 2024	sp. nov.	006445	1
8	*Arthrobacter pullicola* Pallen 2024	sp. nov.	006445	1
8	*Bacillus norwichensis* Pallen 2024	sp. nov.	006445	1
8	*Brevibacterium gallinarum* Pallen 2024	sp. nov.	006445	2
8	*Brevundimonas guildfordensis* Pallen 2024	sp. nov.	006445	2
8	*Cellulomonas avistercoris* Pallen 2024	sp. nov.	006445	2
8	*Clostridium gallinarum* Pallen 2024	sp. nov.	006445	2
8	*Comamonas avium* Pallen 2024	sp. nov.	006445	2
8	*Corynebacterium gallinarum* Pallen 2024	sp. nov.	006445	2
8	*Cytobacillus stercorigallinarum* Pallen 2024	sp. nov.	006445	2
8	*Escherichia whittamii* Pallen 2024	sp. nov.	006445	2
8	*Kaistella pullorum* Pallen 2024	sp. nov.	006445	2
8	*Luteimonas colneyensis* Pallen 2024	sp. nov.	006445	2
8	*Microbacterium commune* Pallen 2024	sp. nov.	006445	2
8	*Microbacterium gallinarum* Pallen 2024	sp. nov.	006445	2
8	*Microbacterium pullorum* Pallen 2024	sp. nov.	006445	2
8	*Oceanitalea stevensii* Pallen 2024	sp. nov.	006445	2
8	*Ochrobactrum gallinarum* Pallen 2024	sp. nov.	006445	2
8	*Oerskovia douganii* Pallen 2024	sp. nov.	006445	2
8	*Oerskovia gallyi* Pallen 2024	sp. nov.	006445	2
8	*Oerskovia merdavium* Pallen 2024	sp. nov.	006445	2
8	*Oerskovia rustica* Pallen 2024	sp. nov.	006445	3
8	*Paenibacillus gallinarum* Pallen 2024	sp. nov.	006445	3
8	‘*Phocaeicola gallinarum*’ Pallen 2024†	sp. nov.	006445	3
8	*Planococcus wigleyi* Pallen 2024	sp. nov.	006445	3
8	*Psychrobacter communis* Pallen 2024	sp. nov.	006445	3
8	*Serpens gallinarum* Pallen 2024	sp. nov.	006445	3
8	*Solibacillus faecavium* Pallen 2024	sp. nov.	006445	3
8	*Sporosarcina gallistercoris* Pallen 2024	sp. nov.	006445	3
8	*Sporosarcina quadrami* Pallen 2024	sp. nov.	006445	3
8	*Stenotrophomonas pennii* Pallen 2024	sp. nov.	006445	3
8	*Ureibacillus galli* Pallen 2024	sp. nov.	006445	3
9	*Maribellus mangrovi* Wang *et al.* 2024	sp. nov.	006448	7
10	*Microbacterium algihabitans* Lee *et al.* 2024	sp. nov.	006443	8
10	*Microbacterium phycohabitans* Lee *et al.* 2024	sp. nov.	006443	8
10	*Microbacterium galbum* Lee *et al.* 2024	sp. nov.	006443	9
11	*Acutalibacter caecimuris* He *et al.* 2024	sp. nov.	006449	11
11	*Acutalibacter intestini* He *et al.* 2024	sp. nov.	006449	11
11	‘*Neglectibacter caecimuris*’ He *et al.* 2024‡	sp. nov.	006449	12
12	*Aquibaculum arenosum* Ahn *et al.* 2024	sp. nov.	006458	5
12	*Aquibaculum* Ahn *et al.* 2024	gen. nov.	006458	7
13	*Noviherbaspirillum album* Li *et al.* 2024	sp. nov.	006450	10
14	*Arthrobacter horti* Park and Kim 2024	sp. nov.	006459	7
15	*Methylobacterium amylolyticum* Zhang *et al.* 2024	sp. nov.	006457	6
15	*Methylobacterium ligniniphilum* Zhang *et al.* 2024	sp. nov.	006457	7
16	*Pedobacter faecalis* Park *et al.* 2024	sp. nov.	006454	7
17	*Lacrimispora brassicae* Kobayashi *et al.* 2024	sp. nov.	006456	6
18	*Phenylobacterium montanum* Tang *et al.* 2024	sp. nov.	006463	6
19	*Sphingobacterium tenebrionis* Zhang *et al.* 2024	sp. nov.	006455	6
20	*Neisseria leonii* Boutroux *et al.* 2024	sp. nov.	006460	11
21	*Cognatishimia coralii* Liu *et al.* 2024	sp. nov.	006467	6
22	*Taklimakanibacter* He *et al.* 2024	gen. nov.	006462	6
22	*Taklimakanibacter deserti* He *et al.* 2024	sp. nov.	006462	6
22	*Taklimakanibacter lacteus* He *et al.* 2024	sp. nov.	006462	8
23	*Flavobacterium adhaerens* Peng *et al.* 2024	sp. nov.	006471	6
23	*Flavobacterium maritimum* Peng *et al.* 2024	sp. nov.	006471	7
24	*Planococcus shenhongbingii* Ning *et al.* 2024	sp. nov.	006465	7
24	*Planococcus shixiaomingii* Ning *et al.* 2024	sp. nov.	006465	7
24	*Planococcus liqunii* Ning *et al.* 2024	sp. nov.	006465	8
25	*Paractinoplanes* Roman *et al.* 2024	gen. nov.	006464	10
25	*Paractinoplanes abujensis* (Sazak *et al.* 2012) Roman *et al.* 2024	comb. nov. [basonym: *Actinoplanes abujensis* Sazak *et al.* 2012]	006464	10
23	*Paractinoplanes aksuensis* (Ding *et al.* 2023) Roman *et al.* 2024	comb. nov. [basonym: *Actinoplanes aksuensis* Ding *et al.* 2023]	006464	11
25	*Paractinoplanes atraurantiacus* (Zhang *et al*. 2012) Roman *et al.* 2024	comb. nov. [basonym: *Actinoplanes atraurantiacus* Zhang *et al*. 2012]	006464	11
25	*Paractinoplanes bogorensis* (Nurkanto *et al.* 2016) Roman *et al.* 2024	comb. nov. [basonym: *Actinoplanes bogorensis* corrig. Nurkanto *et al.* 2016]	006464	11
25	*Paractinoplanes brasiliensis* (Thiemann *et al*. 1969) Roman *et al.* 2024	comb. nov. [basonym: *Actinoplanes brasiliensis* Thiemann *et al*. 1969 (Approved Lists 1980)]	006464	11
25	*Paractinoplanes cibodasensis* (Nurkanto *et al*. 2015) Roman *et al.* 2024	comb. nov. [basonym: *Actinoplanes cibodasensis* Nurkanto *et al*. 2015]	006464	11
25	*Paractinoplanes deccanensis* (Parenti *et al*. 1975) Roman *et al.* 2024	comb. nov. [basonym: *Actinoplanes deccanensis* Parenti *et al*. 1975 (Approved Lists 1980)]	006464	11
25	*Paractinoplanes deserti* (Habib *et al.* 2019) Roman *et al.* 2024	comb. nov. [basonym: *Actinoplanes deserti* Habib *et al.* 2019]	006464	12
25	*Paractinoplanes durhamensis* (Goodfellow *et al*. 1990) Roman *et al.* 2024	comb. nov. [basonym: *Actinoplanes durhamensis* Goodfellow *et al*. 1990]	006464	12
25	*Paractinoplanes ferrugineus* (Palleroni 1979) Roman *et al.* 2024	comb. nov. [basonym: *Actinoplanes ferrugineus* Palleroni 1979 (Approved Lists 1980)]	006464	12
25	*Paractinoplanes globisporus* (Thiemann 1967) Roman *et al.* 2024	comb. nov. [basonym: *Amorphosporangium globisporum* Thiemann 1967 (Approved Lists 1980)]	006464	12
25	*Paractinoplanes hotanensis* (Ding *et al*. 2023) Roman *et al.* 2024	comb. nov. [basonym: *Actinoplanes hotanensis* Ding *et al*. 2023]	006464	12
25	*Paractinoplanes lichenicola* (Saeng-in *et al*. 2021) Roman *et al.* 2024	comb. nov. [basonym: *Actinoplanes lichenicola* Saeng-in *et al*. 2021]	006464	13
25	*Paractinoplanes maris* (Xie *et al*. 2023) Roman *et al.* 2024	comb. nov. [basonym: *Actinoplanes maris* Xie *et al*. 2023]	006464	13
25	*Paractinoplanes ovalisporus* (Saeng-in *et al*. 2021) Roman *et al.* 2024	comb. nov. [basonym: *Actinoplanes ovalisporus* Saeng-in *et al*. 2021]	006464	13
25	*Paractinoplanes polyasparticus* (Ding *et al.* 2023) Roman *et al.* 2024	comb. nov. [basonym: *Actinoplanes polyasparticus* Ding *et al.* 2023]	006464	13
25	*Paractinoplanes pyxinae* (Somphong *et al*. 2024) Roman *et al.* 2024	comb. nov. [basonym: *Actinoplanes pyxinae* Somphong *et al*. 2024]	006464	13
25	*Paractinoplanes ramoplaninifer* (Marcone *et al*. 2017) Roman *et al.* 2024	comb. nov. [basonym: *Actinoplanes ramoplaninifer* Marcone *et al*. 2017]	006464	13
25	*Paractinoplanes rhizophilus* (He *et al*. 2015) Roman *et al.* 2024	comb. nov. [basonym: *Actinoplanes rhizophilus* He *et al*. 2015]	006464	14
25	*Paractinoplanes rishiriensis* (Yamamura *et al*. 2012) Roman *et al.* 2024	comb. nov. [basonym: *Actinoplanes rishiriensis* Yamamura *et al*. 2012]	006464	14
25	*Paractinoplanes sediminis* (Qu *et al*. 2018) Roman *et al.* 2024	comb. nov. [basonym: *Actinoplanes sediminis* Qu *et al*. 2018]	006464	14
25	*Paractinoplanes tereljensis* (Ara *et al.* 2010) Roman *et al.* 2024	comb. nov. [basonym: *Actinoplanes tereljensis* Ara *et al.* 2010]	006464	14
25	*Paractinoplanes toevensis* (Ara *et al*. 2010) Roman *et al.* 2024	comb. nov. [basonym: *Actinoplanes toevensis* Ara *et al*. 2010]	006464	14
25	*Paractinoplanes tropicalis* (Nurkanto *et al.* 2015) Roman *et al.* 2024	comb. nov. [basonym: *Actinoplanes tropicalis* Nurkanto *et al.* 2015]	006464	15
25	*Winogradskya* Roman *et al.* 2024	gen. nov.	006464	15
25	*Winogradskya consettensis* (Goodfellow *et al.* 1990) Roman *et al.* 2024	comb. nov. [basonym: *Actinoplanes consettensis* Goodfellow *et al.* 1990]	006464	15
25	*Winogradskya humida* (Goodfellow *et al*. 1990) Roman *et al.* 2024	comb. nov. [basonym: *Actinoplanes humidus* Goodfellow *et al*. 1990]	006464	15
25	*Symbioplanes* Roman *et al.* 2024	gen. nov.	006464	15
25	*Symbioplanes lichenis* (Phongsopitanun *et al*. 2016) Roman *et al.* 2024	comb. nov. [basonym: *Actinoplanes lichenis* Phongsopitanun *et al*. 2016]	006464	16
25	*Amorphoplanes* Roman *et al.* 2024§	gen. nov.	006464	16
25	*Amorphoplanes auranticolor* (Couch 1963) Roman *et al.* 2024§	comb. nov. [basonym: *Amorphosporangium auranticolor* Couch 1963 (Approved Lists 1980)]	006464	16
25	*Amorphoplanes digitatus* corrig. (Couch 1963) Roman *et al.* 2024§|	comb. nov. [basonym: ‘*Ampullaria digitata*’ Couch 1963]	006464	16
25	*Amorphoplanes friuliensis* (Aretz *et al*. 2001) Roman *et al.* 2024§	comb. nov. [basonym: *Actinoplanes friuliensis* Aretz *et al*. 2001]	006464	16
25	*Amorphoplanes nipponensis* (Wink *et al*. 2014) Roman *et al.* 2024§	comb. nov. [basonym: *Actinoplanes nipponensis* Wink *et al*. 2014]	006464	16
26	*Winogradskyella pelagia* Li *et al.* 2024	sp. nov.	006470	6
27	*Aurantiacibacter hainanensis* Liu *et al.* 2024	sp. nov.	006469	7
27	*Qipengyuania zhejiangensis* Liu *et al.* 2024	sp. nov.	006469	9
28	*Flavobacterium facile* Irgang *et al.* 2024	sp. nov.	006468	6
29	*Pseudomonas beijingensis* Liao *et al.* 2024	sp. nov.	006473	7
30	*Pseudomonas paeninsulae* Ge *et al.* 2024	sp. nov.	006466	11
30	*Pseudomonas svalbardensis* Ge *et al.* 2024	sp. nov.	006466	11
31	*Roseibium algae* Fan *et al.* 2024	sp. nov.	006475	7
32	*Salinibacterium soli* Wan *et al.* 2024	sp. nov.	006479	7
33	*Mesorhizobium retamae* Ben Gaied *et al.* 2024	sp. nov.	006478	8
34	*Collimonas rhizosphaerae* Uroz *et al.* 2024	sp. nov.	006481	9
35	*Methanoculleus frigidifontis* Lai *et al.* 2024	sp. nov.	006477	8
35	*Methanoculleus oceani* Lai *et al.* 2024	sp. nov.	006477	8
35	*Methanoculleus methanifontis* Lai *et al.* 2024	sp. nov.	006477	8
35	*Methanoculleus nereidis* Lai *et al.* 2024	sp. nov.	006477	8
35	*Methanoculleus formosensis* Lai *et al.* 2024	sp. nov.	006477	9
35	*Methanoculleus caldifontis* Lai *et al.* 2024	sp. nov.	006477	9

*Valid publication of the name is based on a single culture collection deposit of the type strain, as approved by the Chair of the ICSP, the Chair of the Judicial Commission and the Editor-in-Chief of the IJSEM.

†According to Rule 31a of the ICNP [2], the name is not validly published. The description refers to a previous description by Gilroy *et al*. in 2021 but therein the name of strain Sa1YUN3 with GenBank assembly accession number GCA_014837055.1 (biosample accession number SAMN15803232) is *Phocaeicola faecium* and not *Phocaeicola gallinarum*. Both *Phocaeicola faecium* and *Phocaeicola gallinarum* were described as *Candidatus* species by Gilroy *et al*. in 2021.

‡‘*Neglectibacter caecimuris*’ He *et al*. 2024 is not validly published because the genus name ‘Neglectibacter’ Zgheib *et al*. 2022 was not validly published at the time of publication. The name *Neglectibacter* Zgheib *et al*. 2022 will be validated in Validation List no. 219 [3].

§Illegitimate name. The genus name *Amorphoplanes* Roman *et al*. 2014 is illegitimate because it is a later homotypic synonym of *Amorphosporangium* Couch 1963 (Approved Lists 1980); see also Rule 51b of the ICNP [2].

|The authors gave *Amorphoplanes digitatis* (Stackebrandt and Kroppenstedt 1988) Roman *et al.* 2024 as comb. nov. with basonym *Actinoplanes digitatis* (Couch 1963) Stackebrandt and Kroppenstedt 1988.

The following taxonomic opinions (i.e., the emendation of circumscriptions or the creation of synonyms) do not affect the status of a name under the International Code of Nomenclature of Prokaryotes. These taxonomic opinions can neither be considered as approved nor as disapproved by the International Committee on Systematics of Prokaryotes.

**Table:** 

Name/authors:	Article no.	Page no.
*Clostridium indicum* Gundawar *et al.* 2019 pro synon. *Lacrimispora amygdalina* (Parshina *et al.* 2003) Haas and Blanchard 2020	006456	6
*Clostridium methoxybenzovorans* Mechichi *et al.* 1999 pro synon. *Lacrimispora indolis* (McClung and McCoy 1957) Haas and Blanchard 2020	006456	6
*Lacrimispora amygdalina* (Parshina *et al.* 2003) Haas and Blanchard 2020 emend. Kobayashi *et al.* 2024	006456	7
*Lentibacter* Li *et al.* 2012 emend. Hahnke *et al.* 2024	006472	10
*Lentibacter algarum* Li *et al.* 2012 emend. Hahnke *et al.* 2024	006472	10
*Noviherbaspirillum* Lin *et al.* 2013 emend. Li *et al.* 2024	006450	10
*Rhizobium indigoferae* Wei *et al.* 2002 pro synon. *Rhizobium leguminosarum* (Frank 1879) Frank 1889 (Approved Lists 1980)	006451	4
*Sinorhizobium kummerowiae* Wei *et al.* 2002 pro synon. *Sinorhizobium meliloti* (Dangeard 1926) De Lajudie *et al.* 1994	006451	4
*Sphingopyxis jiangsuensis* Gao *et al.* 2023 emend. Chen *et al.* 2024*	006446	4
*Sphingopyxis lutea* Chhetri *et al.* 2023 pro synon. *Sphingopyxis jiangsuensis* Gao *et al.* 2023	006446	4

*The authors gave ‘Emended description of *Sphingopyxis jiangsuensis* sp. nov. Gao *et al.* 2023’.

## Correction to Notification that new names of prokaryotes, new combinations, and new taxonomic opinions have appeared in volume 74, part 3 of the IJSEM [4]

*Microbulbifer magnicolonia* Tao *et al.* 2014 should be corrected to *Microbulbifer magnicolonia* Tao *et al.* 2024.

We thank S. Turner (NCBI, NIH, Bethesda, MD, USA) for alerting us to this error.
